# Pure and Hybrid SCAN, rSCAN, and r^2^SCAN: Which One Is Preferred in KS- and HF-DFT Calculations, and How Does D4 Dispersion Correction Affect This Ranking?

**DOI:** 10.3390/molecules27010141

**Published:** 2021-12-27

**Authors:** Golokesh Santra, Jan M. L. Martin

**Affiliations:** Department of Molecular Chemistry and Materials Science, Weizmann Institute of Science, Rehovot 7610001, Israel; gershom@weizmann.ac.il

**Keywords:** HF-DFT, self-consistent, SCAN, rSCAN, r^2^SCAN, D4, regularization

## Abstract

Using the large and chemically diverse GMTKN55 dataset, we have tested the performance of pure and hybrid KS-DFT and HF-DFT functionals constructed from three variants of the SCAN meta-GGA exchange-correlation functional: original SCAN, rSCAN, and r^2^SCAN. Without any dispersion correction involved, HF-SCANn outperforms the two other HF-DFT functionals. In contrast, among the self-consistent variants, SCANn and r^2^SCANn offer essentially the same performance at lower percentages of HF-exchange, while at higher percentages, SCANn marginally outperforms r^2^SCANn and rSCANn. However, with D4 dispersion correction included, all three HF-DFT-D4 variants perform similarly, and among the self-consistent counterparts, r^2^SCANn-D4 outperforms the other two variants across the board. In view of the much milder grid dependence of r^2^SCAN vs. SCAN, r^2^SCAN is to be preferred across the board, also in HF-DFT and hybrid KS-DFT contexts.

## 1. Introduction

In 2001, the “Jacob’s Ladder” was proposed [1] as an organizing principle for the DFT “functional zoo”. Rung one is the LDA [2] (local density approximation, exact for a uniform electron gas). Rung two (GGA or generalized gradient approximation) adds the reduced density gradient (see [3,4,5,6] and references therein). The great improvement in performance of GGA over LDA marked a turning point in the acceptance of DFT as a molecular modeling technique. If one eschews empirical parameters and wishes to design a functional purely from known constraints onto the exact exchange-correlation functional, however, it has been shown [7,8] that GGA intrinsically cannot satisfy all of them: the very popular PBE [9] nonempirical GGA, for instance, only satisfies 11 out of 17 constraints [7,8].

Now, to satisfy additional constraints, one needs to climb up the Jacob’s Ladder to rung three, mGGAs (meta-GGAs, where either the Laplacian or the kinetic energy density are included). In 2015, Sun et al. [10] first succeeded in satisfying all 17 constraints with the nonempirical SCAN (strongly constrained and appropriately normed) mGGA functional. Over the years, several studies have proven SCAN’s broad transferability [11] as well as improved DFT description for different systems, such as metal oxides [12], energetics and structures of different ice and silicon phases [11], high-temperature superconductors [13], liquid, water, and ice [14], and so on.

Despite this notable success, one major shortcoming of SCAN is its numerical instability, which mandates the use of dense integration grids and reduces computational efficiency [15,16]. As a remedy, Bartók and Yates [17] proposed a regularized form, rSCAN, which retains the accuracy of the original mGGA form while improving its numerical stability. However, extensive testing [18,19] has suggested that rSCAN broke some of the constraints the original SCAN was fulfilling and consequently lost the remarkable transferability of the original form; for instance, performance for atomization energies significantly deteriorated. In an attempt to combine the transferability of the original SCAN with the greater numerical stability of rSCAN, Sun et al. [20] have proposed the so-called second version of the regularized SCAN (r^2^SCAN). In a recent full-length preprint [21] expanding upon their original rapid communication [20], Sun and coworkers have proposed a “progression of functionals” (rSCAN, r++SCAN, r^2^SCAN, and r^4^SCAN), where the regularized form finally restores all constraints fulfilled by the original SCAN.

In recent years, Sim, Burke, and coworkers [22] have established the theory of density-corrected density functional theory (DC-DFT), which attempts to separate the errors in DFT calculations into two components: imperfections in the functional itself and errors arising from the self-consistent density evaluated with an imperfect functional. Although the major share can be attributed to the first source, density-driven errors can also be significant, or even crucial, for a variety of systems, e.g., reaction barrier heights, stretched bonds, halogen, and chalcogen binding [22,23,24,25,26,27]. One simple solution is using converged Hartree−Fock densities (HF-DFT) instead of the self-consistent ones for the final evaluation of the exchange-correlation (XC) functional. For more details, the reader is referred to a recent review article by Wasserman et al. [25] and a recent paper in questions-and-answers format by Song et al. [28].

In a recent study [29], we have shown, by means of the large and chemically diverse GMTKN55 benchmark suite (general main-group thermochemistry, kinetics, and noncovalent interactions, 55 problem types [30]), that significantly improved performance can be achieved for pure GGA and meta-GGA (mGGA) HF-DFT functionals, as well as for their hybrids (rung four on Jacob’s Ladder), compared to the self-consistent application of the same functionals. In particular, we found [29] a sizable improvement for HF-SCAN over self-consistent SCAN. This prompted the question if that still would be the case for the regularized SCAN variants rSCAN and r^2^SCAN. This question is addressed in the present study.

Our objective is to investigate which SCAN variant offers the best performance in pure as well as hybrid meta-GGA forms of self-consistent and HF-DFT functionals. Both the dispersion uncorrected and D4 [31,32] dispersion corrected functionals are taken into account.

## 2. Computational Methods

All the electronic structure calculations involving rSCAN and r^2^SCAN mGGA XC-functionals have been performed using the ORCA [33] 5.0.1 package running on the Faculty of Chemistry HPC facility. The DEFGRID3 integration grid has been used throughout. Results for pure and hybrid HF-DFT and self-consistent SCAN are extracted from our previous work [29], where we used ORCA 4.2.1 with GRID 6. The def2-QZVPP [34] basis set was employed throughout, except for five anionic subsets: MB16-43, HEAVY28, HEAVYSB11, ALK8, CHB6, and ALKBDE10, where we employed def2-QZVPPD [35] instead. This basis set combination, which is very close to the basis set limit for mGGA and hybrid KS-DFT functionals, is a de facto standard for this type of benchmark calculation. See, for example, [30,36].

Grimme group’s GMTKN55 [30] database has been used for this entire study. Total 55 subsets can be further divided into five top-level subcategories: small molecule thermochemistry, barrier heights, intermolecular interactions, conformers (or intra-molecular interactions), and reaction energies for large systems. See Table 1 in [30] for the detailed description and references for all 55 subsets. The WTMAD2 (so-called second version of the weighted mean absolute deviation), as defined in [30], has been used as the primary metric throughout the present study:

(1) $$WTMAD2=\frac{1}{\sum_{i=1}^{55}N_{i}}\;.\;\overset{55}{\underset{i=1}{\sum }}N_{i}.\;\frac{56.84\;\mathrm{kcal}/\mathrm{mol}}{{\bar{\left|∆E\right|}}_{i}}\;\cdot \;MAD_{i}\;$$

where ${\bar{\left|∆E\right|}}_{i}$ is the mean absolute value of all the reference energies from i=1 to 55, $N_{i}$ is the number of systems in each subset, and $MAD_{i}$ is the mean absolute difference between calculated and reference energies for each of the 55 subsets.

Now, for the functionals, we have used the same terminology we proposed in [29] for different GGAs and mGGAs—HF-SCAN*n*, HF-rSCAN*n*, and HF-r^2^SCAN*n* for the HF-DFT series and SCAN*n*, rSCAN*n*, and r^2^SCAN*n* for the corresponding self-consistent counterparts, where n is the percentage of HF exchange (HFx) used for the hybrid form, and it ranges from 0–50%.

The D4 [31,32] dispersion correction is used throughout to check the effect of using empirical dispersion correction and whether it alters the performance ranking of different SCAN variants. We refitted s_8_, a_1_, and a_2_ for each functional by minimizing WTMAD2 over the full GMTKN55 (two other parameters, s_6_ and the prefactor for the three-body Axilrod−Teller−Muto [37,38] correction, c_ATM_, were kept fixed at unity throughout). All the original and the refitted D4 parameters are listed in Table S1 in Supplementary Materials. We note in passing that in a recent study, Ehlert et al. [39] have reported a set of D4 parameters only for the self-consistent rSCAN and r^2^SCAN pure mGGA functionals. However, the set used by Ehlert et al. for optimizing the D4 parameters is different from what we have used in this study. So, we reoptimized the D4 parameters for both the rSCAN and r^2^SCAN to compare *apples to apples*.

Powell’s derivative-free constrained optimizer, BOBYQA [40] (bound optimization by quadratic approximation), and a collection of scripts developed in-house were used to optimize all the parameters.

Burke and coworkers [28] pointed out that results with SCAN and HF-SCAN for the G21IP subset did not yet seem to be converged in terms of grid size, even for GRID6 and GRID7. We explored grid sensitivity for that subset, as well as for its companion G21EA and, on account of its very large weight in WTMAD2 formula, for the RG18 subset. The results can be found in Figures S1–S3 of the Supplementary Materials. In our previous work [41] on the DSD-SCAN double hybrids, we explored grid convergence in both the radial and angular grids and found the former to be much more crucial than the latter; in that work, we established convergence with an unpruned 590-point Lebedev angular grid [42] and a 150 Euler–Maclaurin radial grid [43,44]. The GRID6 in ORCA4 corresponds to a pruned 590-point angular grid—which is hence adequate—but a much coarser radial grid. The number of points in the latter can be increased by manually setting the “IntAcc” keyword: according to Equation (14) in [45], every unit of IntAcc adds 15 radial points. After some initial experimentation, we settled on IntAcc = 8 rather than the default of 5.34 and additionally used IntAcc = 10 to confirm convergence. For G21IP, the remaining difference between GRID6 and GRID6, IntAcc = 8 is at most 2.0 kcal/mol (0.09 eV), and the mean absolute deviation (MAD) is 0.7 kcal/mol (0.03 eV). Considering also the very small weight factor 0.221 of the G21IP set in the WTMAD2 formula, we deem the GRID6 results adequately converged for our purposes. Nevertheless, for practical applications of the HF-SCAN and SCAN variants, we still recommend increasing IntAcc to 8. For rSCAN and r^2^SCAN, in contrast, we saw no indication that grids finer than DEFGRID3 would be necessary.

## 3. Results and Discussion

### 3.1. Without Dispersion Correction

**Figure 1:**
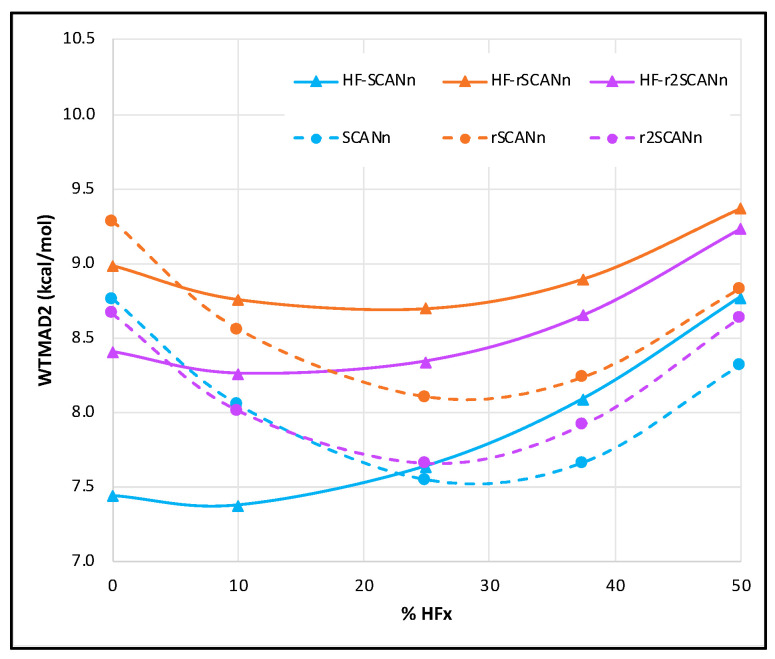
WTMAD2 (kcal/mol) trend with respect to the percentage of HF-like exchange (n) for self-consistent and HF-DFT forms of SCAN, rSCAN, and r^2^SCAN. The solid lines represent the HF-DFTs, and the dashed lines are for the corresponding self-consistent series.

Among the three variants of the self-consistent series, rSCANn and r^2^SCANn offer similar performance at a smaller percentage of HF-exchange (HFx), and at a higher percentage of HFx, SCANn marginally outperforms r^2^SCANn. Identical to the self-consistent SCANn [29], the overall WTMAD2 minimum is near 30% HFx for both the rSCANn and r^2^SCANn. Among the three HF-DFT series, HF-SCANn wins the race, followed by HF-r^2^SCANn. As long as the pure mGGA is concerned, the WTMAD2 gaps between different SCAN variants are most significant, and they decrease with the increase of *n* value. For HF-SCANn and HF-r^2^SCANn, the WTMAD2 minimum is near 10% HFx, which shifts to ~25% for HF-rSCANn. The WTMAD2 values of pure mGGA and hybrid forms with 10% HFx are very close to each other for these three HF-DFT series in hand (see Figure 1).

**Figure 2:**
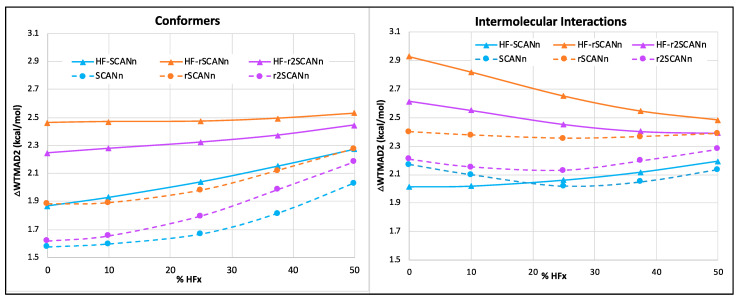
Trend of WTMAD2 contribution (ΔWTMAD2 in kcal/mol) with respect to the percentage of HF-like exchange for the intermolecular interaction and conformer subsets. The solid lines represent the HF-DFTs, and the dashed lines are for the corresponding self-consistent series.

Among the five top-level subsets, for the conformers, HF-SCANn and SCANn emerge as the best in class among the HF-DFT and self-consistent series, respectively. Irrespective of the choice of SCAN variant, the self-consistent forms always perform better than their HF-DFT counterparts. Hybrid functionals are similar or worse performers compared to the pure mGGA forms. SCAN and r^2^SCAN are the two best functionals one can choose for conformers (see Figure 2). For the intermolecular interactions, the choice of SCAN variant significantly affects the performance of HF-DFT, particularly at the small percentage of HFx. Here too, the HF-SCANn and SCANn are better performers than their two regularized HF-DFT and self-consistent forms. Next, for small molecule thermochemistry, barrier heights, and reaction energies of large molecules, the choice of SCAN variant has very little to no effect on the performance of HF-DFT and self-consistent series (see Figure S4 in Supplementary Materials).

Interestingly enough, the SCANn series only surpasses the performance of HF-SCANn beyond 22% HFx, while for rSCANn and r^2^SCANn, this crossover happens near 5%. Significantly better performance of the self-consistent rSCANn and r^2^SCANn series than their HF-DFT counterparts for the intermolecular interactions and conformers is the reason behind this “early crossover” (See Table S2 in Supplementary Materials for the breakdown of total WTMAD2 into five major subcategories for all the functionals).

We are now shifting our focus to individual subsets of GMTKN55 that are most affected by using different SCAN variants (see Figure S5 in Supplementary Materials). For HAL59, the HF-SCANn series offers significantly better performance than HF-rSCANn and HF-r^2^SCANn. The use of HF-orbitals only benefits the regular SCAN variant, up to 30% HFx. For the interaction energies of pnictogen-containing dimers (PNICO23) and WATER27, the choice of SCAN variant only matters for the pure mGGA and hybrid functionals with a small percentage of HFx. The same is true for the large organic molecule isomerization (ISOL24), where the original SCAN variants are better than rSCAN and r^2^SCAN, both for the self-consistent and HF-DFT series.

The self-consistent forms consistently surpass the HF-DFT functionals for amino acid conformers (AMINO20x4), and the use of rSCAN or r^2^SCAN instead of the original SCAN results in more harm than good for the HF-DFT series. For 1,4-butanediol, HF-SCANn is the best pick among the three HF-DFT series. However, among the self-consistent series, both rSCANn and r^2^SCANn outperform SCANn at a lower percentage of HFx.

The S66 [46,47] noncovalent interaction subset is, in fact, a mixture of different types of interactions and can be further divided into four subgroups: systems 1 through 23 are hydrogen bonds, 24–33 are π-stacking, systems 34–46 are London dispersion complexes, and the remainder as mixed-influence. For London dispersion, mixed, and π-stacking, the HF-SCANn and SCANn are the best performers among the HF-DFT and self-consistent DFT series, respectively. For the H-bonds, the HF-SCANn series performs significantly better than two other HF-DFT series at a lower percentage of HFx. On the other hand, self-consistent rSCANn and r^2^SCANn are better performers than SCANn throughout (see Table S3 in Supplementary Materials)

Ref. [28] points out that the open-shell species in such subsets as BH76 (barrier heights) and RSH43 (radical separation energies) are affected by spin contamination. What if we use restricted open-shell Hartree–Fock (ROHF) and ditto Kohn–Sham (ROKS) densities for HF-DFT and the self-consistent series, respectively? This causes only 0.11 and 0.12 kcal/mol improvements of WTMAD2 for HF-r^2^SCAN and r^2^SCAN, respectively, and even less for the other two variants of HF-DFT and self-consistent functionals. However, this gain increases to about 0.2 and 0.4 kcal/mol for *hybrid* HF-DFT and self-consistent functionals, respectively (see Table S2 in Supplementary Materials). If we look at individual subsets, for all three pure mGGA HF-DFT functionals, performance improves for RSE43 and deteriorates for SIE4x4. Next, for all three hybrid HF-DFT functionals, the most significant gain is for W4-11, and switching from the original to any of the two regularized SCAN variants is beneficial for the BH76 subset. For all three self-consistent pure mGGA functionals, we see the most significant improvement for BH76, which becomes more pronounced for hybrid functionals. (See Table S4 in Supplementary Materials for individual subsets).

### 3.2. Impact of Introducing D4 Dispersion Correction

**Figure 3:**
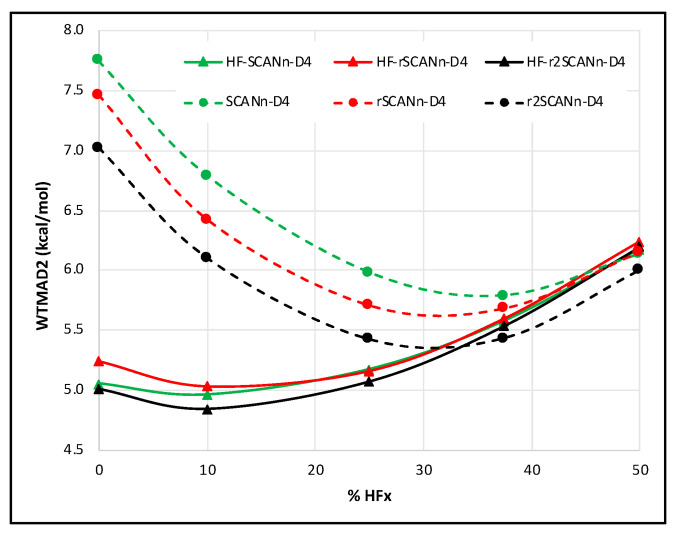
WTMAD2 (kcal/mol) trend with respect to the percentage of HF-like exchange (n) for self-consistent and HF-DFT-D4 forms of SCAN, rSCAN, and r^2^SCAN. The solid lines represent the HF-DFTs, and the dashed lines are for the corresponding self-consistent series.

Considering D4 dispersion correction on top of the HF-SCANn, HF-rSCANn, HF-r^2^SCANn, and their respective self-constant counterparts improve WTMAD2 throughout. The HF-DFT series draw the most benefit. All three dispersion-corrected self-consistent series have an overall minimum near 38% HFx. Among them, r^2^SCANn-D4 is the best pick for all values of n. However, the WTMAD2 gap between r^2^SCANn-D4 and SCANn-D4 is relatively tiny, near 50% HFx. Unlike for the dispersion-uncorrected cases, the choice of SCAN variant has practically no effect on the performance of the three HF-DFT-D4 series (see Figure 3). The same as HF-SCANn-D4 [29], both the HF-rSCANn-D4 and HF-r^2^SCANn-D4 have the overall WTMAD2 minimum near 10% HFx.

Interestingly, r^2^SCANn-D4 marginally outperforms the HF-r^2^SCANn-D4 series beyond 35% HFx, and the other two self-consistent series approach their HF-DFT counterparts only near 50%.

**Figure 4:**
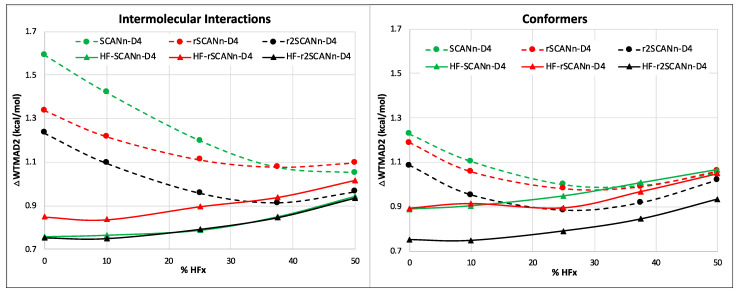
Trend of WTMAD2 contribution (ΔWTMAD2 in kcal/mol) with respect to the percentage of HF-like exchange for the intermolecular interaction and conformer subsets. The solid lines represent the HF-DFT-D4 series, and the dashed lines are for the corresponding self-consistent series.

Among the five top-level subsets, for small molecule thermochemistry and barrier heights, the performance trends for different SCAN variants are similar to what we observed for dispersion uncorrected cases. For the large molecule reactions, the choice of SCAN variant seems to matter, mainly for the hybrid HF-DFT and self-consistent functionals. For conformers, HF-r^2^SCANn-D4 and r^2^SCANn-D4 are the two best picks among the HF-DFT and self-consistent series, respectively. In the case of the intermolecular interactions, the HF-SCANn-D4 series is surpassed by both the HF-r^2^SCANn-D4 and HF-rSCANn-D4. Among the self-consistent series, r^2^SCANn-D4 is still the best performer (see Figure 4). Both for the conformers and intermolecular interactions, HF-DFT-D4 always offers better or equal performance when compared to their self-consistent counterparts. (See Table S1 in Supplementary Materials for the breakdown of total WTMAD2 into five major subsets).

Now, we look into the most affected subsets of GMTKN55 in detail (see Figure S6 in Supplementary Materials). In general, the performance difference we obtained for various subsets using different variants of dispersion-uncorrected HF-DFT is now gone as soon as we include D4 dispersion correction.

Among the self-consistent series, r^2^SCANn-D4 and rSCANn-D4 marginally outperform the rSCANn-D4 for HAL59. For the rare gas clusters (RG18), both HF-rSCANn-D4 and HF-r^2^SCANn-D4 perform similarly and are better choices than the HF-SCANn-D4 series. Among the three self-consistent series, hybrid r^2^SCAN and rSCAN functionals win the race for this particular subset. For 1,4-butanediol, r^2^SCAN is the preferred choice among the three SCAN variants, both for the pure and hybrid self-consistent mGGA functionals with a small percentage of HFx.

Once again, we divide the mixed bag S66 noncovalent interactions into four subcategories: hydrogen bonds, π-stacking, London dispersion, and the mixed-influence (see Table 1). The rSCAN variant is the best in class among HF-DFT and the self-consistent series for the London dispersion and the mixed-influence. All the self-consistent SCAN variants perform similarly for π-stacking, whereas HF-r^2^SCANn-D4 is always inferior to HF-rSCANn-D4 and HF-SCANn-D4. For the hydrogen bonds, r^2^SCANn-D4 is the best performer among the self-consistent series, and HF-r^2^SCANn-D4 is the best among the HF-DFT series. Without considering dispersion correction, all three SCAN variants, either self-consistent or density-corrected, largely underbind π-stacking, London dispersion, and mixed subsets, which become slightly better by introducing D4 correction. Except for the dispersion-uncorrected HF-DFT cases, every other functional overbinds hydrogen bonds.

## 4. Conclusions

From a comprehensive study of self-consistent and density-corrected pure and hybrid SCAN, rSCAN, and r^2^SCAN mGGA functionals, we can conclude the following:

- Both for self-consistent and for HF-DFT series, the WTMAD2 global minimum is the same for all three SCAN variants. The only exception is HF-rSCANn, where the overall minimum is near 25% HF exchange instead of near 10% for the other two. Among all the functionals tested, the pure mGGA form is a low-cost alternative for all three SCAN variants.
- The choice of SCAN variant can significantly influence the performance of the dispersion-uncorrected HF-DFT series. However, upon introducing D4 dispersion, the WTMAD2 gaps between different SCAN variants almost vanish. At lower percentages of HF exchange, self-consistent SCANn and r^2^SCANn hybrids perform similarly. However, with D4 correction, r^2^SCANn-D4 outperforms rSCANn-D4 and SCANn-D4.
- For the small-molecule thermochemistry and barrier height subsets, different SCAN variants perform comparably in the pure and hybrid self-consistent and HF-DFT series.
- Irrespective of the choice of SCAN variant, the use of ROHF and ROKS densities are clearly beneficial for hybrid HF-DFT and self-consistent functionals.
- Among all the functionals tested, HF-r^2^SCAN_10_-D4 offers the lowest WTMAD2 (4.85 kcal/mol), just below HF-SCAN_10_-D4 (WTMAD2 = 4.96 kcal/mol) and without the latter’s grid convergence issues. The same remark applies concerning HF-DFT, with HF-r^2^SCAN-D4 (WTMAD2 = 5.01 kcal/mol) slightly outperforming HF-SCAN-D4 (WTMAD2 = 5.05 kcal/mol).
- Overall, and taking into account the reduced grid sensitivity resulting from its regularization, we find that r^2^SCAN’s superiority over SCAN is also retained for hybrids and for HF-DFT.

## Figures and Tables

**Table 1:** MAD and MSD (mean absolute and mean signed deviations, kcal/mol) of HF-DFT-D4 and KS-DFT-D4 functionals for the S66 subset and for four subcategories of S66.

Functionals	MAD (kcal/mol)					MSD (kcal/mol)				
	H-Bonds	π-Stack	London	Mixed-Influence	Full S66	H-Bonds	π-Stack	London	Mixed Influence	Full S66
HF-SCAN-D4	0.21	0.57	0.47	0.23	0.32	0.09	0.57	−0.45	0.02	0.03
HF-SCAN_10_-D4	0.31	0.44	0.44	0.24	0.33	0.24	0.44	−0.42	0.07	0.09
HF-SCAN0-D4	0.45	0.26	0.41	0.25	0.35	0.42	0.26	−0.41	0.11	0.14
HF-SCAN_38_-D4	0.59	0.15	0.37	0.28	0.39	0.58	0.15	−0.37	0.16	0.2
HF-SCAN_50_-D4	0.79	0.13	0.28	0.32	0.45	0.79	0.13	−0.26	0.26	0.32
SCAN-D4	0.73	0.1	0.34	0.23	0.41	0.73	−0.03	−0.34	0.01	0.19
SCAN_10_-D4	0.79	0.08	0.23	0.2	0.39	0.79	−0.01	−0.22	0.1	0.26
SCAN0-D4	0.84	0.08	0.16	0.23	0.41	0.84	−0.02	−0.14	0.18	0.32
SCAN_38_-D4	0.89	0.1	0.14	0.27	0.43	0.89	−0.05	−0.11	0.22	0.35
SCAN_50_-D4	0.98	0.1	0.11	0.32	0.48	0.98	−0.03	−0.06	0.29	0.42
HF-rSCAN-D4	0.11	0.52	0.26	0.16	0.21	0.03	0.52	−0.24	0.01	0.05
HF-rSCAN_10_-D4	0.18	0.38	0.28	0.15	0.22	0.15	0.38	−0.27	0.03	0.07
HF-rSCAN0-D4	0.43	0.33	0.22	0.22	0.31	0.43	0.33	−0.21	0.15	0.20
HF-rSCAN_38_-D4	0.59	0.16	0.24	0.24	0.35	0.59	0.16	−0.24	0.17	0.23
HF-rSCAN_50_-D4	0.80	0.11	0.19	0.30	0.42	0.80	0.10	−0.18	0.26	0.34
rSCAN-D4	0.57	0.19	0.13	0.15	0.30	0.56	−0.17	−0.13	−0.04	0.13
rSCAN_10_-D4	0.63	0.14	0.08	0.13	0.30	0.63	−0.10	−0.07	0.04	0.20
rSCAN0-D4	0.74	0.10	0.06	0.16	0.33	0.74	−0.04	−0.01	0.14	0.29
rSCAN_38_-D4	0.84	0.11	0.07	0.22	0.39	0.84	−0.04	0.00	0.21	0.35
rSCAN_50_-D4	0.97	0.13	0.07	0.29	0.46	0.97	−0.04	0.00	0.28	0.42
HF-r^2^SCAN-D4	0.14	0.77	0.40	0.25	0.32	−0.09	0.77	−0.35	0.06	0.03
HF-r^2^SCAN_10_-D4	0.16	0.75	0.33	0.28	0.32	0.13	0.75	−0.27	0.16	0.15
HF-r^2^SCAN0-D4	0.37	0.58	0.28	0.30	0.36	0.36	0.58	−0.23	0.22	0.24
HF-r2SCAN_38_-D4	0.52	0.34	0.30	0.30	0.38	0.52	0.34	−0.28	0.22	0.24
HF-r^2^SCAN_50_-D4	0.72	0.21	0.26	0.33	0.43	0.72	0.21	−0.25	0.27	0.32
r^2^SCAN-D4	0.47	0.09	0.33	0.20	0.30	0.44	−0.04	−0.33	−0.05	0.07
r^2^SCAN_10_-D4	0.51	0.09	0.27	0.19	0.30	0.50	−0.03	−0.27	0.01	0.12
r^2^SCAN0-D4	0.62	0.08	0.20	0.24	0.34	0.62	0.00	−0.19	0.17	0.23
r^2^SCAN_38_-D4	0.73	0.08	0.16	0.24	0.37	0.73	0.00	−0.14	0.18	0.28
r^2^SCAN_50_-D4	0.88	0.08	0.12	0.29	0.43	0.88	0.02	-0.09	0.27	0.37

## Data Availability

Data are available in Electronic Supporting Information (ESI), and for additional details, please contact the authors.

## Supplementary Materials

The following are available online, Table S1: original and optimized D4 parameters, total WTMAD2 (kcal/mol), as well as its decomposition into the five major subsets for HF-DFT and self-consistent functionals; Table S2: total WTMAD2 (kcal/mol) and its decomposition into the five major subsets for dispersion uncorrected HF-DFT and self-consistent functionals; Table S3: MAD and MSD (mean absolute and mean signed deviations, kcal/mol) of dispersion-uncorrected HF-DFT and KS-DFT functionals for the S66 subset, and the four subcategories of S66; Table S4: effect of using ROHF and ROKS densities instead of UHF and UKS ones for HF-DFT and self-consistent pure mGGA and hybrid functionals. Green means improvement and red means deterioration of performance; Figure S1: energy difference (in kcal/mol) for all 36 ionization potentials of G21IP subset with different grid choices. We have used the energies evaluated using GRID6 and IntAcc = 10 as our reference; Figure S2: energy difference (in kcal/mol) for all 25 electron affinities of G21EA subset with different grid choices. We have used the energies evaluated using GRID6 and IntAcc = 10 as our reference; Figure S3: energy difference for 18 interaction energies of RG18 subset with different grid choices. We have used the energies evaluated using GRID6 and IntAcc = 10 as our reference; Figure S4: the trend of WTMAD2 contribution (ΔWTMAD2) (Y-axis) with respect to the percentage of HF exchange (X-axis) for three top-level subsets of GMTKN55 (namely, small molecule thermochemistry; barrier heights and reaction energies for large systems) in case of both dispersion uncorrected (left) and corrected (right) series; Figure S5: dependence of WTMAD2 (kcal/mol) contribution (Y-axis) on the percentage of HF exchange (X-axis) for the dispersion uncorrected HF-DFT and the self-consistent series for the individual subsets SIE4x4, WATER27, BH76, RG18, W4-11, BHPERI, S66, PX13, HAL59, PNICO23, ADIM6, IDISP, alkane conformers (ACONF), 1,4-butanediol conformers (BUT14DIOL), oligopeptide conformers (PCONF21), sugar conformers (SCONF), amino acid conformers (AMINO20X4), TAUT15, G21EA, DC13, and large-molecule isomerization (ISOL24) subsets; Figure S6: dependence of WTMAD2 (kcal/mol) contribution (Y-axis) on the percentage of HF exchange (X-axis) for self-consistent and HF-DFT-D4 series for the individual subsets SIE4x4, WATER27, BH76, RG18, W4-11, BHPERI, S66, PX13, HAL59, PNICO23, ADIM6, IDISP, alkane conformers (ACONF), 1,4-butanediol conformers (BUT14DIOL), oligopeptide conformers (PCONF21), sugar conformers (SCONF), amino acid conformers (AMINO20X4), TAUT15, G21EA, DC13, and large-molecule isomerization (ISOL24) subsets.
