# Supplementary material for: Pure and Hybrid SCAN, rSCAN, and r2SCAN: Which One Is Preferred in KS- and HF-DFT Calculations, and How Does D4 Dispersion Correction Affect This Ranking?
Source: Molecules. 2021 Dec 27;27(1):141. doi: 10.3390/molecules27010141 (PMC8746565; doi:10.3390/molecules27010141)
Supplement: Supplementary file 1 [file molecules-27-00141-s001.zip › molecules-1498462-supplementary.pdf]

Electronic Supporting Information
for

Pure and hybrid SCAN, rSCAN, and r2SCAN: which one is preferred in KS- and HF-DFT calculations, and how does D4 dispersion correction affect this ranking?

Golokesh Santra and Jan M.L. Martin*

Department of Molecular Chemistry and Materials Science, Weizmann Institute of Science, 7610001 Reḥovot, Israel.

Correspondence: golokesh.santra@weizmann.ac.il

Table S1: Original and optimized D4 parameters, total WTMAD2 (kcal/mol), as well as its decomposition into the five major subsets for HF-DFT and self-consistent functionals.

Functionals	s_6	s_8	c_{ATM}	a_1	a_2	WTMAD2	THERMO[a]	BARRIER[b]	LARGE[c]	CONF[d]	INTERMOL[e]
HF-SCAN-D4	1.0	0.6726	1.0	0.1654	7.3664	5.05	1.286	1.040	1.080	0.890	0.757
HF-SCAN$_{10}$-D4	1.0	1.0159	1.0	0.2161	7.3021	4.96	1.334	0.898	1.059	0.905	0.764
HF-SCAN0-D4	1.0	1.0482	1.0	0.2290	7.2144	5.17	1.509	0.786	1.141	0.949	0.787
HF-SCAN$_{38}$-D4	1.0	0.8788	1.0	0.2493	6.9488	5.58	1.702	0.767	1.254	1.008	0.849
HF-SCAN$_{50}$-D4	1.0	0.5959	1.0	0.2810	6.4527	6.18	1.947	0.849	1.374	1.067	0.943
SCAN-D4orig[1,2]	1.0	1.4613	1.0	0.6293	6.3128	7.87	1.682	1.900	1.347	1.237	1.708
SCAN-D4	1.0	3.6151	1.0	0.2044	9.0611	7.75	1.681	1.895	1.360	1.227	1.592
SCAN$_{10}$-D4	1.0	6.4925	1.0	0.3149	8.7697	6.79	1.531	1.510	1.228	1.105	1.419
SCAN0-D4	1.0	6.1187	1.0	0.3750	8.1124	5.98	1.549	1.040	1.192	1.001	1.198
SCAN$_{38}$-D4	1.0	5.0438	1.0	0.3996	7.6249	5.79	1.691	0.768	1.256	0.997	1.075
SCAN$_{50}$-D4	1.0	3.2856	1.0	0.4108	6.9783	6.14	1.901	0.754	1.377	1.055	1.051
HF-rSCAN-D4	1.0	0.2691	1.0	0.2634	5.7949	5.25	1.323	1.098	1.083	0.893	0.848
HF-rSCAN$_{10}$-D4	1.0	0.3824	1.0	0.2672	5.9709	5.04	1.391	0.950	0.946	0.914	0.836
HF-rSCAN0-D4	1.0	0.1534	1.0	0.2400	5.8491	5.16	1.573	0.860	0.942	0.895	0.895
HF-rSCAN$_{38}$-D4	1.0	0.1986	1.0	0.2425	6.0003	5.60	1.776	0.842	1.078	0.968	0.938
HF-rSCAN$_{50}$-D4	1.0	0.2520	1.0	0.2867	5.8287	6.24	2.035	0.895	1.248	1.049	1.015
rSCAN-D4orig[3]	1.0	0.8773	1.0	0.4911	5.7586	7.57	1.649	1.889	1.423	1.196	1.418
rSCAN-D4	1.0	3.6523	1.0	0.3926	7.2936	7.46	1.647	1.873	1.419	1.189	1.337
rSCAN$_{10}$-D4	1.0	3.3927	1.0	0.4012	7.0515	6.43	1.483	1.505	1.163	1.058	1.217
rSCAN0-D4	1.0	2.9139	1.0	0.4131	6.7174	5.71	1.537	1.044	1.035	0.982	1.111
rSCAN$_{38}$-D4	1.0	2.4737	1.0	0.4170	6.4918	5.68	1.721	0.798	1.098	0.991	1.077
rSCAN$_{50}$-D4	1.0	1.7038	1.0	0.4161	6.1499	6.15	1.969	0.792	1.235	1.061	1.097
HF-r^{2}SCAN-D4	1.0	0.1596	1.0	0.0915	6.8013	5.01	1.290	1.014	0.969	0.986	0.753
HF-r^{2}SCAN$_{10}$-D4	1.0	0.0364	1.0	0.1074	6.4775	4.85	1.376	0.901	0.898	0.920	0.750
HF-r^{2}SCAN0-D4	1.0	0.0765	1.0	0.1529	6.3025	5.07	1.572	0.818	0.977	0.914	0.792
HF-r2SCAN$_{38}$-D4	1.0	0.0827	1.0	0.1695	6.3400	5.54	1.782	0.816	1.121	0.975	0.845
HF-r^{2}SCAN$_{50}$-D4	1.0	0.1265	1.0	0.1891	6.2827	6.20	2.047	0.885	1.284	1.049	0.934
r^{2}SCAN-D4orig[3]	1.0	0.6019	1.0	0.5156	5.7734	7.23	1.552	1.809	1.314	1.123	1.427
r^{2}SCAN-D4	1.0	2.9784	1.0	0.2428	8.2088	7.02	1.554	1.793	1.355	1.087	1.235
r^{2}SCAN$_{10}$-D4	1.0	3.1930	1.0	0.2825	7.8993	6.10	1.450	1.433	1.172	0.953	1.095
r^{2}SCAN0-D4	1.0	3.1720	1.0	0.3221	7.4820	5.42	1.540	0.983	1.062	0.883	0.956
r^{2}SCAN$_{38}$-D4	1.0	2.5473	1.0	0.3396	7.0745	5.43	1.725	0.755	1.125	0.918	0.912
r^{2}SCAN$_{50}$-D4	1.0	1.7036	1.0	0.3559	6.5555	6.00	1.980	0.777	1.261	1.021	0.963

[a]THERMO=Small Molecule Thermochemistry; [b]BARRIER=barrier heights; [c]LARGE=reaction energies for large systems; [d]CONF=conformer/intramolecular interactions; and [e]INTERMOL=intermolecular interactions

Table S2: Total WTMAD2 (kcal/mol) and its decomposition into the five major subsets for dispersion uncorrected HF-DFT and self-consistent functionals.

Functionals	Densities	THERMO[a]	BARRIER[b]	LARGE[c]	CONF[d]	INTERMOL[e]	WTMAD2
HF-SCAN	UHF	1.287	0.983	1.288	1.869	2.014	7.44
HF-SCAN	ROHF	1.297	0.967	1.231	1.869	2.014	7.38
HF-$SCAN_{10}$	UHF	1.331	0.846	1.252	1.930	2.019	7.38
HF-SCAN0	UHF	1.496	0.739	1.304	2.041	2.060	7.64
HF-SCAN0	ROHF	1.348	0.739	1.310	2.041	2.060	7.50
HF-$SCAN_{38}$	UHF	1.683	0.738	1.403	2.153	2.116	8.09
HF-$SCAN_{50}$	UHF	1.920	0.859	1.529	2.273	2.192	8.77
SCAN	UKS	1.674	1.865	1.481	1.576	2.169	8.77
SCAN	ROKS	1.732	1.749	1.452	1.580	2.173	8.69
$SCAN_{10}$	UKS	1.522	1.476	1.357	1.598	2.098	8.05
SCAN0	UKS	1.540	0.995	1.322	1.668	2.020	7.55
SCAN0	ROKS	1.351	0.812	1.254	1.673	2.026	7.12
$SCAN_{38}$	UKS	1.672	0.734	1.391	1.815	2.048	7.66
$SCAN_{50}$	UKS	1.875	0.762	1.516	2.030	2.134	8.32
HF-rSCAN	UHF	1.339	0.990	1.271	2.463	2.925	8.99
HF-rSCAN	ROHF	1.383	0.995	1.201	2.463	2.9254	8.97
HF-$rSCAN_{10}$	UHF	1.393	0.861	1.219	2.470	2.817	8.76
HF-rSCAN0	UHF	1.555	0.786	1.236	2.473	2.650	8.70
HF-rSCAN0	ROHF	1.420	0.743	1.214	2.473	2.650	8.50
HF-$rSCAN_{38}$	UHF	1.745	0.808	1.305	2.494	2.544	8.90
HF-$rSCAN_{50}$	UHF	1.997	0.915	1.444	2.531	2.483	9.37
rSCAN	UKS	1.647	1.817	1.535	1.883	2.400	9.28
rSCAN	ROKS	1.698	1.726	1.531	1.883	2.4001	9.24
$rSCAN_{10}$	UKS	1.477	1.442	1.373	1.892	2.377	8.56
rSCAN0	UKS	1.519	0.984	1.270	1.981	2.353	8.11
rSCAN0	ROKS	1.347	0.817	1.222	1.981	2.353	7.72
$rSCAN_{38}$	UKS	1.688	0.767	1.298	2.121	2.365	8.24
$rSCAN_{50}$	UKS	1.932	0.818	1.416	2.277	2.388	8.83
HF-r^2SCAN	UHF	1.316	0.923	1.310	2.247	2.613	8.41
HF-r^2SCAN	ROHF	1.276	0.922	1.240	2.247	2.613	8.30
HF-r^2SCAN_{10}	UHF	1.380	0.807	1.248	2.279	2.549	8.26
HF-r^2SCAN0	UHF	1.559	0.747	1.265	2.324	2.451	8.35
HF-r^2SCAN0	ROHF	1.402	0.705	1.245	2.324	2.451	8.13
HF-$r2SCAN_{38}$	UHF	1.754	0.782	1.343	2.373	2.402	8.65
HF-r^2SCAN_{50}	UHF	2.011	0.907	1.481	2.445	2.390	9.23
r^2SCAN	UKS	1.559	1.749	1.536	1.618	2.207	8.67
r^2SCAN	ROKS	1.549	1.653	1.528	1.618	2.207	8.55
r^2SCAN_{10}	UKS	1.452	1.383	1.369	1.655	2.152	8.01
r^2SCAN0	UKS	1.530	0.934	1.266	1.797	2.129	7.65
r^2SCAN0	ROKS	1.315	0.758	1.236	1.786	2.116	7.21
r^2SCAN_{38}	UKS	1.698	0.728	1.309	1.985	2.195	7.92
r^2SCAN_{50}	UKS	1.946	0.802	1.431	2.183	2.278	8.64

[a]THERMO=Small Molecule Thermochemistry; [b]BARRIER=barrier heights; [c]LARGE=reaction energies for large systems; [d]CONF=conformer/intramolecular interactions; and [e]INTERMOL=intermolecular interactions

Table S3: MAD and MSD (mean absolute and mean signed deviations, kcal/mol) of dispersion-uncorrected HF-DFT and KS-DFT functionals for the S66 subset, and the four subcategories of S66.

**Functionals**	**MAD (kcal/mol)**					**MSD (kcal/mol)**				
	**H-bonds**	**π-stack**	**London**	**Mixed-influence**	**Ful S66**	**H-bonds**	**π-stack**	**London**	**Mixed-influence**	**Full S66**
HF-SCAN	0.51	1.38	2.06	0.94	1.08	-0.45	-1.38	-2.06	-0.94	-1.06
HF-SCAN$_{10}$	0.43	1.52	2.06	0.91	1.06	-0.32	-1.52	-2.06	-0.90	-1.02
HF-SCAN0	0.39	1.75	2.08	0.91	1.09	-0.15	-1.75	-2.08	-0.88	-0.99
HF-SCAN$_{38}$	0.42	1.94	2.09	0.91	1.13	-0.01	-1.94	-2.09	-0.87	-0.97
HF-SCAN$_{50}$	0.49	2.13	2.11	0.92	1.19	0.13	-2.13	-2.11	-0.85	-0.95
SCAN	0.57	1.24	1.45	0.71	0.89	0.40	-1.24	-1.45	-0.62	-0.53
SCAN$_{10}$	0.57	1.44	1.56	0.74	0.95	0.38	-1.44	-1.56	-0.66	-0.59
SCAN0	0.57	1.73	1.72	0.80	1.04	0.36	-1.73	-1.72	-0.72	-0.70
SCAN$_{38}$	0.59	1.96	1.83	0.84	1.12	0.36	-1.96	-1.83	-0.76	-0.76
SCAN$_{50}$	0.63	2.18	1.93	0.88	1.20	0.38	-2.18	-1.93	-0.79	-0.82
HF-rSCAN	0.84	2.26	2.43	1.36	1.52	-0.84	-2.26	-2.43	-1.36	-1.52
HF-rSCAN$_{10}$	0.67	2.30	2.40	1.29	1.45	-0.67	-2.30	-2.40	-1.29	-1.45
HF-rSCAN0	0.48	2.37	2.35	1.19	1.35	-0.42	-2.37	-2.35	-1.19	-1.33
HF-rSCAN$_{38}$	0.41	2.43	2.30	1.12	1.30	-0.22	-2.43	-2.30	-1.11	-1.23
HF-rSCAN$_{50}$	0.44	2.49	2.26	1.06	1.30	-0.01	-2.49	-2.26	-1.02	-1.14
rSCAN	0.38	2.16	1.89	1.05	1.15	0.01	-2.16	-1.89	-1.05	-1.01
rSCAN$_{10}$	0.39	2.25	1.95	1.05	1.18	0.02	-2.25	-1.95	-1.05	-1.04
rSCAN0	0.42	2.39	2.02	1.05	1.22	0.08	-2.39	-2.02	-1.03	-1.05
rSCAN$_{38}$	0.48	2.48	2.07	1.04	1.27	0.15	-2.48	-2.07	-1.01	-1.04
rSCAN$_{50}$	0.55	2.51	2.10	1.03	1.30	0.24	-2.31	-2.10	-0.97	-0.97
HF-r^{2}SCAN	0.83	1.73	2.32	1.16	1.36	-0.83	-1.73	-2.32	-1.16	-1.36
HF-r^{2}SCAN$_{10}$	0.68	1.84	2.30	1.12	1.31	-0.68	-1.84	-2.30	-1.12	-1.31
HF-r^{2}SCAN0	0.49	2.01	2.27	1.06	1.25	-0.44	-2.01	-2.27	-1.06	-1.23
HF-r2SCAN$_{38}$	0.41	2.15	2.25	1.02	1.22	-0.24	-2.15	-2.25	-1.01	-1.16
HF-r^{2}SCAN$_{50}$	0.43	2.28	2.23	1.00	1.24	-0.05	-2.28	-2.23	-0.96	-1.09
r^{2}SCAN	0.37	1.67	1.78	0.89	1.00	-0.01	-1.67	-1.78	-0.88	-0.87
r^{2}SCAN$_{10}$	0.37	1.82	1.86	0.92	1.05	0.00	-1.82	-1.86	-0.90	-0.91
r^{2}SCAN0	0.40	1.85	1.96	0.95	1.09	0.05	-1.65	-1.96	-0.92	-0.90
r^{2}SCAN$_{38}$	0.46	2.21	2.02	0.96	1.18	0.11	-2.21	-2.02	-0.92	-0.97
r^{2}SCAN$_{50}$	0.52	2.37	2.08	1.02	1.26	0.20	-2.37	-2.08	-0.86	-0.96

Table S4: Effect of using ROHF and ROKS densities instead of UHF and UKS ones for HF-DFT and self-consistent pure mGGA and hybrid functionals. Green means improvement and red means deterioration of performance.

Subsets	Δ WTMAD2 ROHF	Δ WTMAD2 UHF	ΔΔ WTMAD2	Δ WTMAD2 ROHF	Δ WTMAD2 UHF	ΔΔ WTMAD2	Δ WTMAD2 ROKS	Δ WTMAD2 UKS	ΔΔ WTMAD2	Δ WTMAD2 ROKS	Δ WTMAD2 UKS	ΔΔ WTMAD2
	HF-r2SCAN			HF-r2SCAN0			r2SCAN			r2SCAN0		
ALKBDE10	0.0163	0.0181	-0.002	0.0255	0.0316	-0.006	0.0198	0.0191	0.001	0.023	0.0272	-0.004
BH76RC	0.1287	0.1482	-0.020	0.1243	0.1631	-0.039	0.1498	0.1657	-0.016	0.126	0.1558	-0.030
BH76	0.4292	0.4305	-0.001	0.3701	0.4121	-0.042	1.0144	1.1112	-0.097	0.3719	0.5418	-0.170
DC13	0.0823	0.0805	0.002	0.078	0.0826	-0.005	0.0712	0.0772	-0.006	0.066	0.0751	-0.009
G21EA	0.1044	0.1292	-0.025	0.1277	0.1598	-0.032	0.087	0.1082	-0.021	0.1143	0.1402	-0.026
G21IP	0.021	0.0236	-0.003	0.0217	0.0254	-0.004	0.0209	0.0246	-0.004	0.0206	0.0253	-0.005
HEAVYSB11	0.0219	0.04	-0.018	0.0261	0.0492	-0.023	0.0244	0.0284	-0.004	0.0199	0.0422	-0.022
INV24	0.0392	0.039	0.000	0.0487	0.0487	0.000	0.034	0.034	0.000	0.0427	0.0427	0.000
MB16-43	0.0384	0.0356	0.003	0.0428	0.0479	-0.005	0.043	0.0442	-0.001	0.0475	0.0482	-0.001
RC21	0.0792	0.0584	0.021	0.075	0.0519	0.023	0.1095	0.1039	0.006	0.0894	0.0911	-0.002
RSE43	0.1334	0.2063	-0.073	0.1555	0.1704	-0.015	0.3259	0.3323	-0.006	0.1326	0.1697	-0.037
SIE4X4	0.2995	0.2203	0.079	0.1657	0.1452	0.021	0.3204	0.3247	-0.004	0.1766	0.1953	-0.019
W4-11	0.0581	0.1179	-0.060	0.2002	0.276	-0.076	0.1127	0.0666	0.046	0.127	0.1995	-0.073
YBDE18	0.0383	0.0535	-0.015	0.0531	0.069	-0.016	0.0447	0.0528	-0.008	0.0383	0.0545	-0.016
	HF-rSCAN			HF-rSCAN0			rSCAN			rSCAN0		
ALKBDE10	0.016	0.0162	0.000	0.0201	0.0258	-0.006	0.0252	0.0209	0.004	0.019	0.0232	-0.004
BH76RC	0.1456	0.1558	-0.010	0.1268	0.1571	-0.030	0.1708	0.1727	-0.002	0.1303	0.1525	-0.022
BH76	0.4695	0.465	0.004	0.3877	0.4302	-0.043	1.0628	1.1538	-0.091	0.4108	0.5775	-0.167
DC13	0.0899	0.0873	0.003	0.0819	0.086	-0.004	0.0753	0.081	-0.006	0.0677	0.0765	-0.009
G21EA	0.1004	0.1207	-0.020	0.1189	0.1463	-0.027	0.0917	0.1003	-0.009	0.106	0.1277	-0.022
G21IP	0.0224	0.0245	-0.002	0.0215	0.0243	-0.003	0.0219	0.0249	-0.003	0.0206	0.0242	-0.004
HEAVYSB11	0.0207	0.0365	-0.016	0.0241	0.0461	-0.022	0.025	0.0267	-0.002	0.019	0.0397	-0.021
INV24	0.0441	0.0437	0.000	0.0489	0.0486	0.000	0.0369	0.0369	0.000	0.0425	0.0425	0.000
MB16-43	0.0399	0.0372	0.003	0.0485	0.052	-0.004	0.0468	0.047	0.000	0.0572	0.0547	0.003
RC21	0.0828	0.0614	0.021	0.0784	0.0555	0.023	0.1165	0.1106	0.006	0.0957	0.0971	-0.001
RSE43	0.1425	0.2158	-0.073	0.1493	0.1684	-0.019	0.3843	0.3884	-0.004	0.158	0.2077	-0.050
SIE4X4	0.3049	0.2233	0.082	0.1698	0.1474	0.022	0.3256	0.3293	-0.004	0.1816	0.1998	-0.018
W4-11	0.0709	0.0719	-0.001	0.1553	0.2273	-0.072	0.1762	0.1084	0.068	0.0965	0.152	-0.056
YBDE18	0.0334	0.0445	-0.011	0.0483	0.0639	-0.016	0.0435	0.0466	-0.003	0.0307	0.0467	-0.016
	HF-SCAN			HF-SCAN0			SCAN			SCAN0		
ALKBDE10	0.0188	0.0199	-0.001	0.0249	0.0309	-0.006	0.0207	0.0192	0.002	0.0189	0.0222	-0.003
BH76RC	0.1405	0.1575	-0.017	0.1435	0.1785	-0.035	0.1728	0.1885	-0.016	0.1455	0.1771	-0.032
BH76	0.4022	0.4185	-0.016	0.3551	0.3555	0.000	1.0282	1.1484	-0.120	0.3802	0.5661	-0.186
DC13	0.0719	0.0717	0.000	0.0778	0.0834	-0.006	0.059	0.0668	-0.008	0.0713	0.0817	-0.010
G21EA	0.093	0.1139	-0.021	0.116	0.149	-0.033	0.0735	0.0949	-0.021	0.1033	0.1324	-0.029
G21IP	0.0208	0.0243	-0.004	0.0214	0.0252	-0.004	0.0558	0.024	0.032	0.0184	0.0224	-0.004
HEAVYSB11	0.0154	0.0271	-0.012	0.021	0.0389	-0.018	0.0162	0.0175	-0.001	0.0134	0.0329	-0.020
INV24	0.0396	0.0393	0.000	0.053	0.053	0.000	0.0574	0.0573	0.000	0.0474	0.0473	0.000
MB16-43	0.0343	0.0353	-0.001	0.0495	0.0507	-0.001	0.06	0.0565	0.004	0.0624	0.0579	0.005
RC21	0.0685	0.0489	0.020	0.0671	0.0511	0.016	0.1422	0.13	0.012	0.1044	0.1104	-0.006
RSE43	0.1448	0.2009	-0.056	0.1772	0.1699	0.007	0.2713	0.3064	-0.035	0.0957	0.1718	-0.076
SIE4X4	0.2919	0.247	0.045	0.1579	0.1468	0.011	0.3136	0.3189	-0.005	0.1704	0.19	-0.020
W4-11	0.0879	0.0813	0.007	0.1405	0.2004	-0.060	0.1793	0.1205	0.059	0.093	0.1399	-0.047
YBDE18	0.0249	0.0318	-0.007	0.032	0.0461	-0.014	0.0504	0.0446	0.006	0.0207	0.0363	-0.016

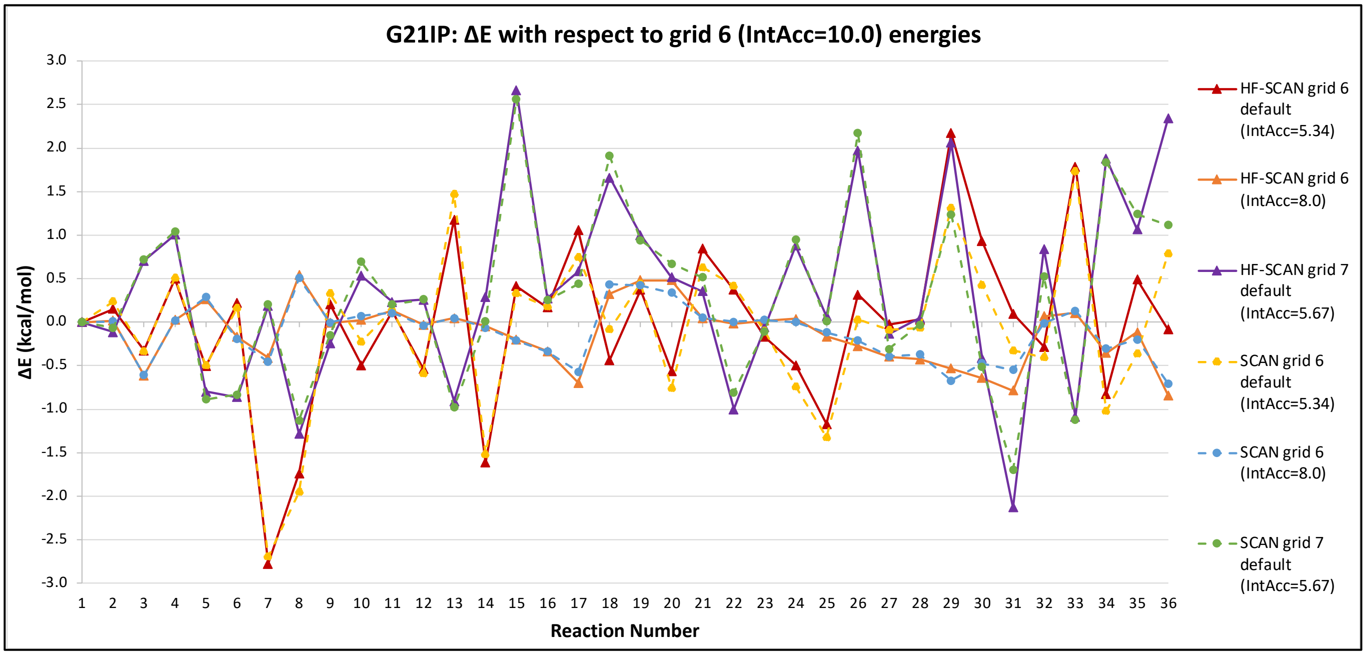

Figure S1: Energy difference (in kcal/mol) for all 36 ionization potentials of G21IP subset with different grid choices. We have used the energies evaluated using GRID6 and IntAcc=10 as our reference.

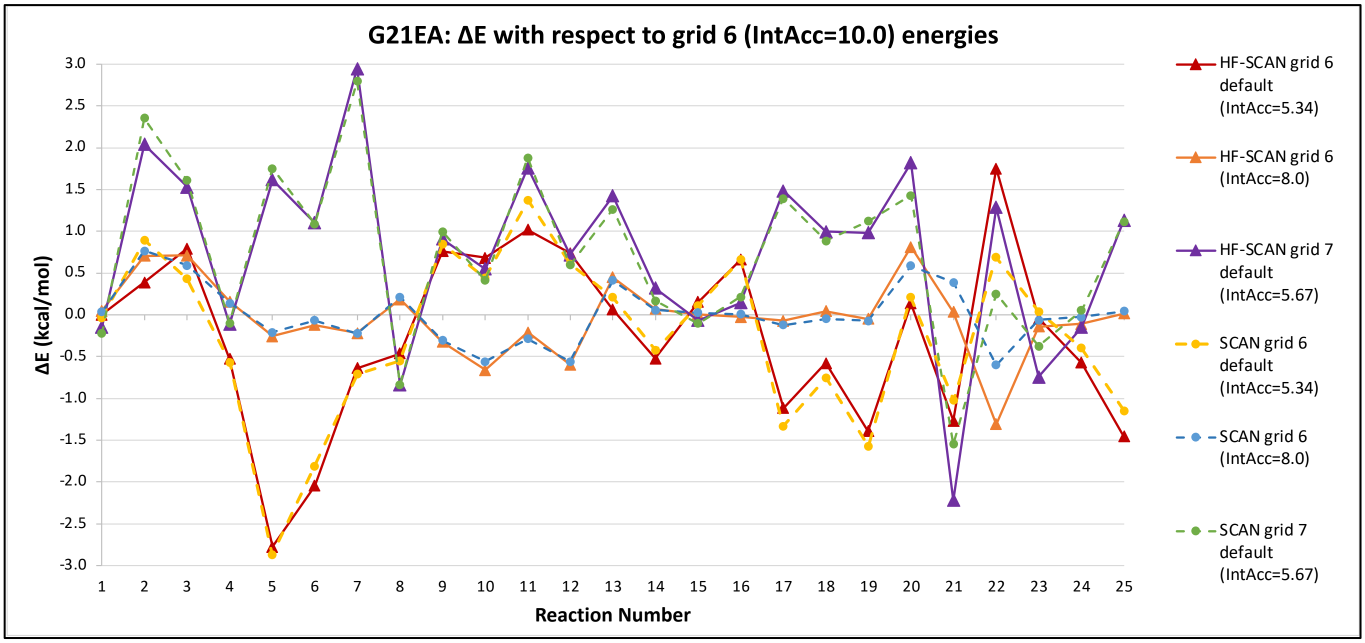

Figure S2: Energy difference (in kcal/mol) for all 25 electron affinities of G21EA subset with different grid choices. We have used the energies evaluated using GRID6 and IntAcc=10 as our reference.

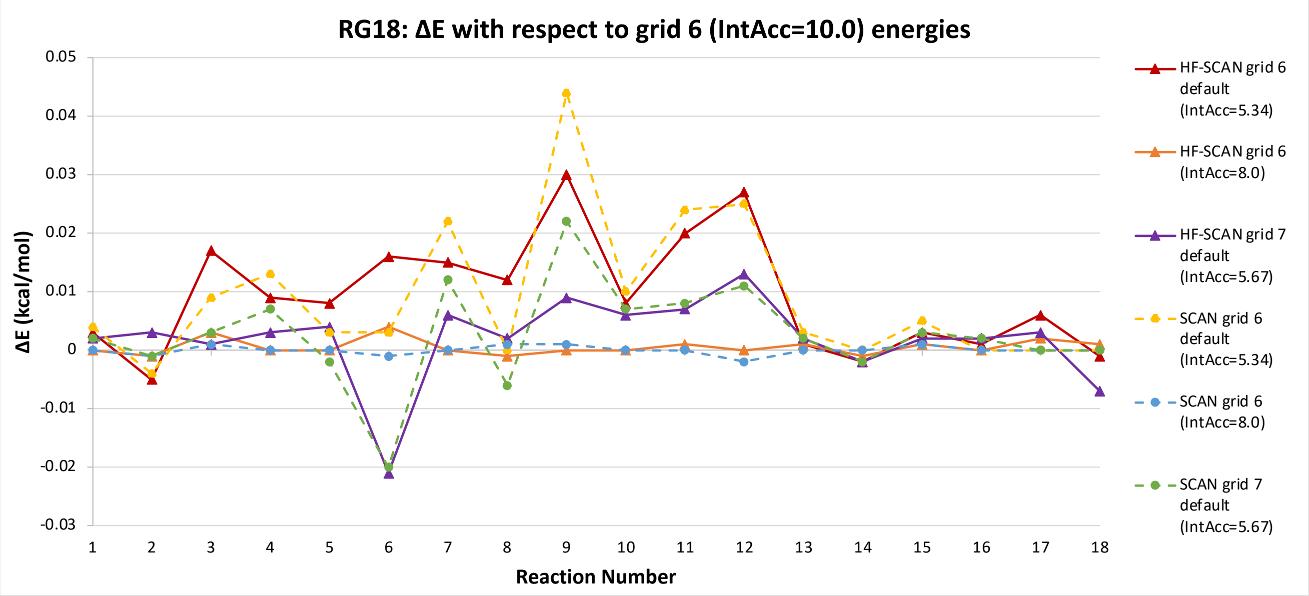

Figure S3: Energy difference for 18 interaction energies of RG18 subset with different grid choices. We have used the energies evaluated using GRID6 and IntAcc=10 as our reference.

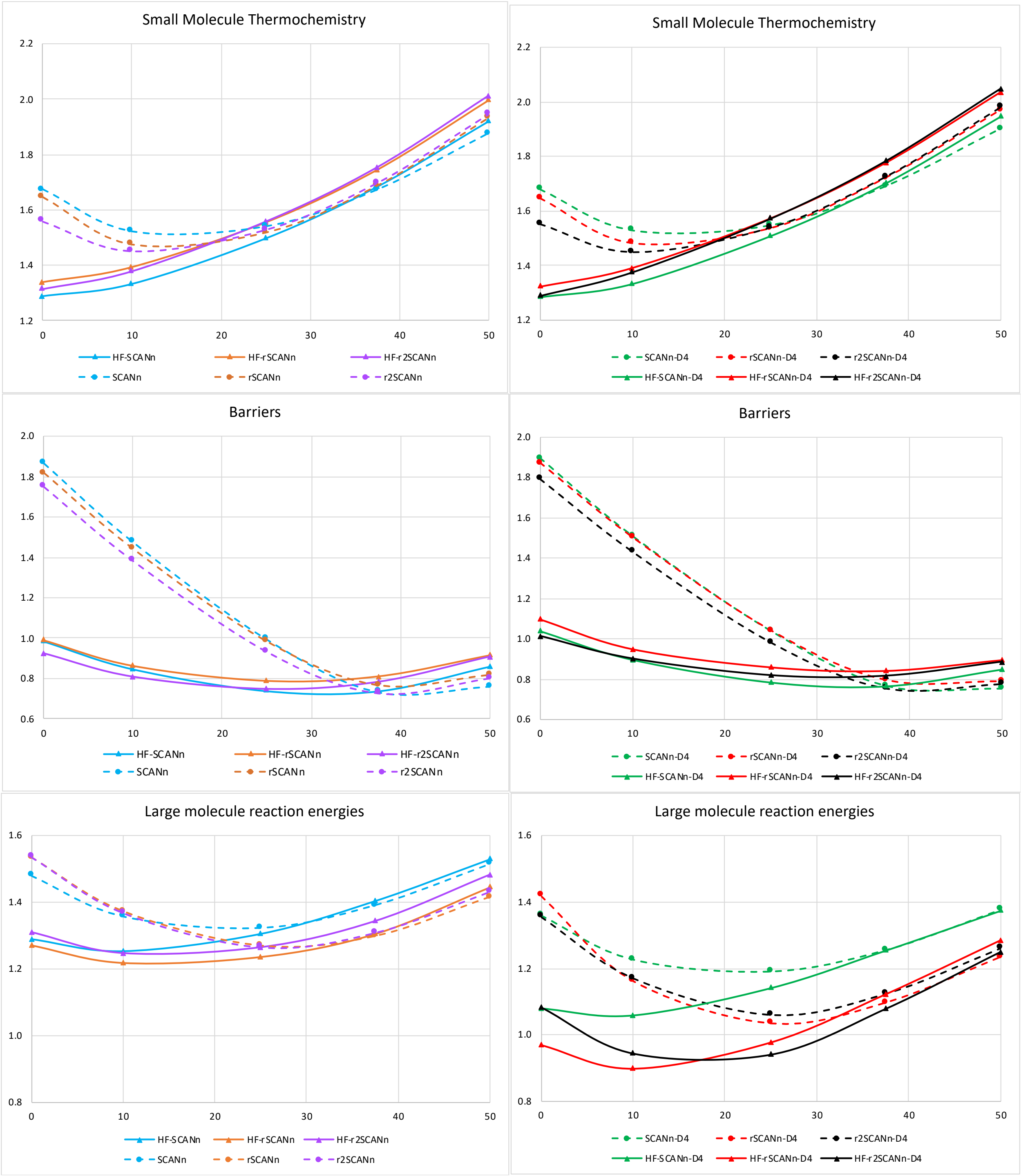

Figure S4: The trend of WTMAD2 contribution (ΔWTMAD2) (Y-axis) with respect to the percentage of HF exchange (X-axis) for three top-level subsets of GMTKN55 (namely, Small Molecule Thermochemistry; barrier heights and reaction energies for large systems) in case of both dispersion uncorrected (left) and corrected(right) series.

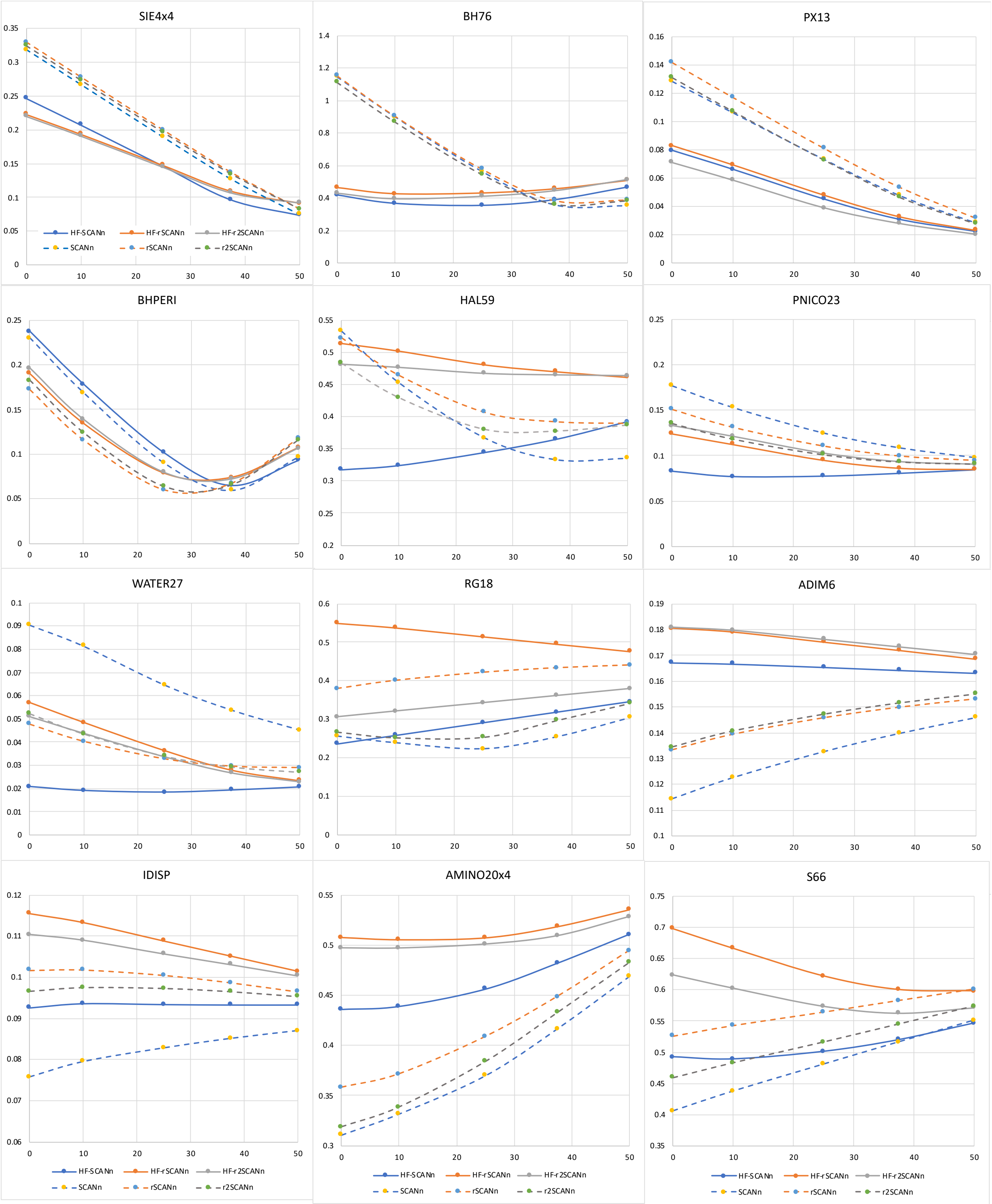

Figure S5: continued

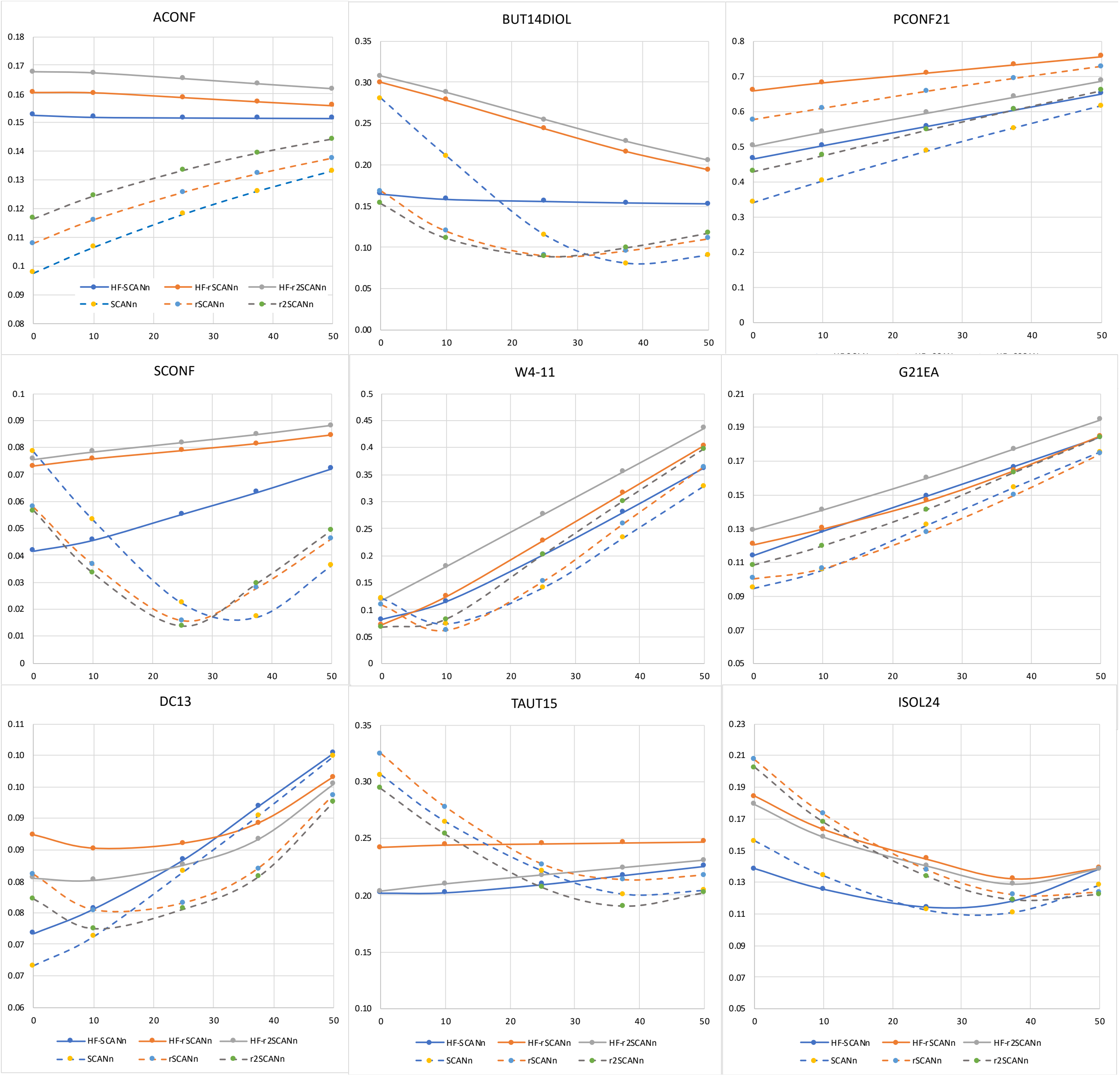

Figure S5: Dependence of WTMAD2 (kcal/mol) contribution (Y-axis) on the percentage of HF exchange (X-axis) for the dispersion uncorrected HF-DFT and the self-consistent series for the individual subsets SIE4x4, WATER27, BH76, RG18, W4-11, BHPERI, S66, PX13, HAL59, PNICO23, ADIM6, IDISP, alkane conformers (ACONF), 1,4-butanediol conformers (BUT14DIOL), oligopeptide conformers (PCONF21), sugar conformers (SCONF), amino acid conformers (AMINO20X4), TAUT15, G21EA, DC13, and large-molecule isomerization (ISOL24) subsets.

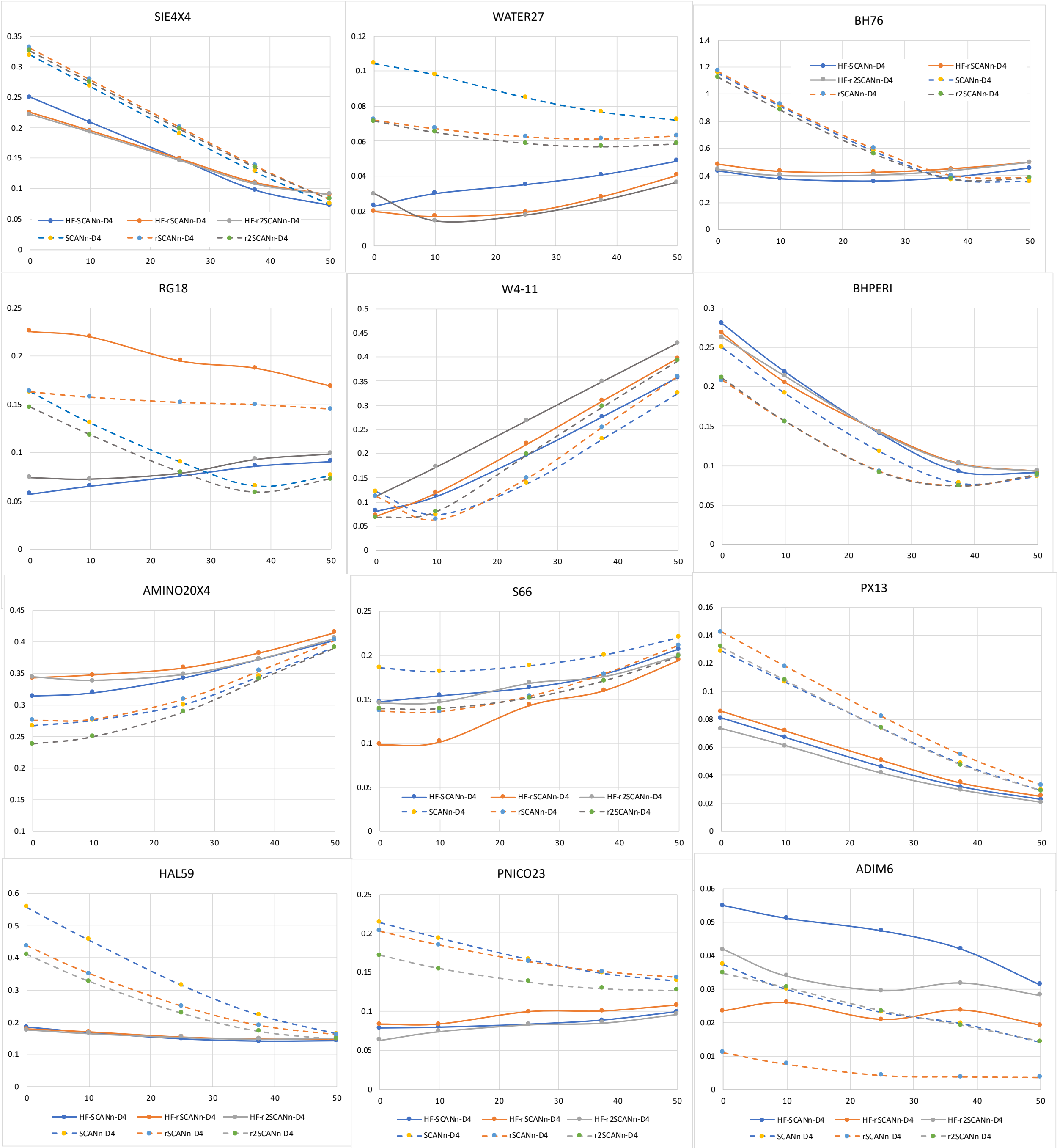

Figure S6: continued

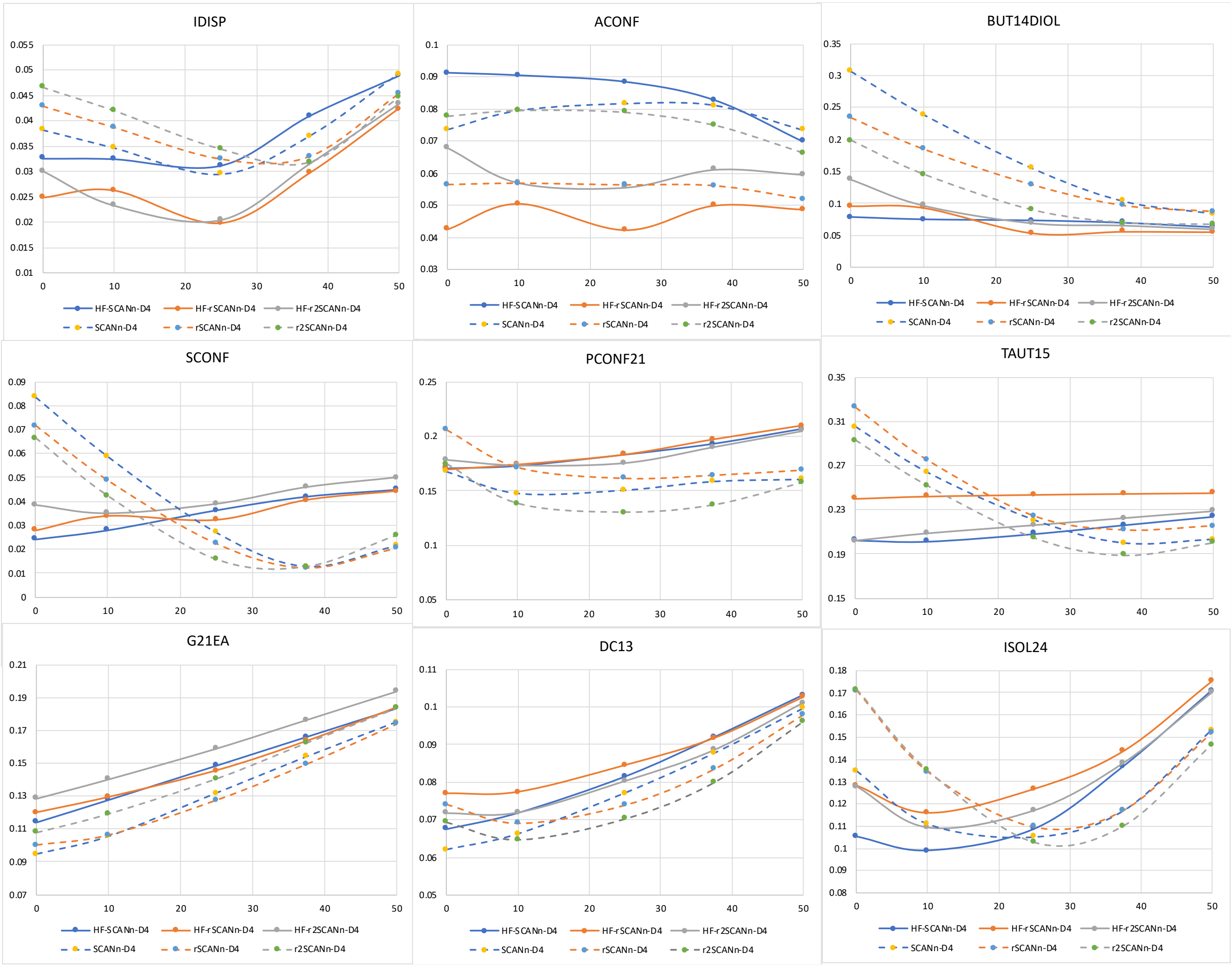

Figure S6: Dependence of WTMAD2 (kcal/mol) contribution (Y-axis) on the percentage of HF exchange (X-axis) for self-consistent and HF-DFT-D4 series for the individual subsets SIE4x4, WATER27, BH76, RG18, W4-11, BHPERI, S66, PX13, HAL59, PNICO23, ADIM6, IDISP, alkane conformers (ACONF), 1,4-butanediol conformers (BUT14DIOL), oligopeptide conformers (PCONF21), sugar conformers (SCONF), amino acid conformers (AMINO20X4), TAUT15, G21EA, DC13, and large-molecule isomerization (ISOL24) subsets.

References:

1. Caldeweyher, E.; Ehlert, S.; Hansen, A.; Neugebauer, H.; Spicher, S.; Bannwarth, C.; Grimme, S. A generally applicable atomic-charge dependent London dispersion correction. *J. Chem. Phys.* **2019**, *150*, 154122, doi:10.1063/1.5090222.
2. Caldeweyher, E.; Bannwarth, C.; Grimme, S. Extension of the D3 dispersion coefficient model. *J. Chem. Phys.* **2017**, *147*, 034112, doi:10.1063/1.4993215.
3. Ehlert, S.; Huniar, U.; Ning, J.; Furness, J.W.; Sun, J.; Kaplan, A.D.; Perdew, J.P.; Brandenburg, J.G. r 2 SCAN-D4: Dispersion corrected meta-generalized gradient approximation for general chemical applications. *J. Chem. Phys.* **2021**, *154*, 061101, doi:10.1063/5.0041008.
